# Bax is essential for mitochondrion-mediated apoptosis but not for cell death caused by photodynamic therapy

**DOI:** 10.1038/sj.bjc.6601298

**Published:** 2003-10-14

**Authors:** S-M Chiu, L-Y Xue, J Usuda, K Azizuddin, N L Oleinick

**Affiliations:** 1 Department of Radiation Oncology and The CWRU/Ireland Comprehensive Cancer Center, School of Medicine (BRB-324), Case Western Reserve University, 10900 Euclid Avenue, Cleveland, OH 44106-4942, USA

**Keywords:** photodynamic therapy, phthalocyanine Pc 4, Bax, apoptosis, cell death

## Abstract

The role of Bax in the release of cytochrome *c* from mitochondria and the induction of apoptosis has been demonstrated in many systems. Using immunocytochemical staining, we observed that photodynamic therapy (PDT) with the photosensitiser Pc 4 induced Bax translocation from the cytosol to mitochondria, and the release of cytochrome *c* from mitochondria as early signalling for the intrinsic pathway of apoptosis in human breast cancer MCF-7c3 cells. To test the role of Bax in apoptosis, MCF-7c3 cells were treated with Bax antisense oligonucleotides, which resulted in as much as a 50% inhibition of PDT-induced apoptosis. In the second approach, Bax-negative human prostate cancer DU-145 cells were studied. Following PDT, the hallmarks of apoptosis, including the release of cytochrome *c* from mitochondria, loss of mitochondrial membrane potential, caspase activation, and chromatin condensation and fragmentation, were completely blocked in these cells. Restoration of Bax expression in DU-145 cells restored apoptosis, indicating that the resistance of DU-145 cells to PDT-induced apoptosis is due to the lack of Bax rather than to another defect in the apoptotic machinery. However, despite the inhibition of apoptosis, the Bax-negative DU-145 cells were as photosensitive as Bax-replete MCF-7c3 cells, as determined by clonogenic assay. Thus, for Pc 4-PDT, the commitment to cell death occurs prior to Bax activation.

Apoptosis, or programmed cell death, is a genetically regulated cellular suicide mechanism essential for multicellular organisms to remove damaged or unwanted cells and maintain tissue homeostasis (Ellis *et al*, 1991; Kroemer *et al*, 1997). Apoptosis can also be induced by external stimuli. The process includes an ordered cascade of enzymatic events leading to the production of unique morphological and biochemical features. Among the most important regulators of the process are members of the Bcl-2 family of proteins (Gross *et al*, 1999; Antonsson and Martinou, 2000; Tsujimoto and Shimizu, 2000). While members such as Bcl-2 and Bcl-xL suppress apoptosis, other members such as Bax, Bak and Bid promote it. Among proapoptotic Bcl-2 family members, Bax is perhaps the best-studied protein. In healthy cells, Bax is located in the cytoplasm, but during apoptosis, it translocates to the mitochondria. This process is probably a consequence of the exposure of its C-terminal membrane-seeking domain that is facilitated by unknown cytosolic factors or by an increase in cellular pH (Wolter *et al*, 1997; Khaled *et al*, 1999; Nomura *et al*, 1999). Whereas in the cytosol Bax exists as monomers, the mitochondrion-inserted Bax is present as dimers and higher oligomers (Eskes *et al*, 2000). The membrane insertion and oligomerisation of Bax is essential for the release of cytochrome *c* and apoptosis, as evidenced by the blockage of apoptosis in Bax mutants that have lost the capacity for mitochondrion insertion due to deletion of the mitochondrion-targeting C-terminus (Nechushtan *et al*, 1999). In addition, overexpression of Bax protein in mammalian cells results in the induction of apoptosis through the release of cytochrome *c* and activation of caspases (Rosse *et al*, 1998; Finucane *et al*, 1999; Gross *et al*, 1999), and purified Bax protein is capable of triggering the release of cytochrome *c* from isolated mitochondria (Xiang *et al*, 1996; Vander Heiden *et al*, 1997; Eskes *et al*, 1998; Jurgensmeier *et al*, 1998; Marzo *et al*, 1998; Narita *et al*, 1998). Although the mechanism by which Bax triggers cytochrome *c* release is not clear, studies in cell-free systems have shown that Bax interacts either with the mitochondrial permeability transition (PT) pore components, the voltage-dependent anion channel (Shimizu *et al*, 1999) and the adenine nucleotide translocator (Marzo *et al*, 1998), or with cardiolipin of the outer membrane (Kuwana *et al*, 2002) to form megachannels that allow the passage of cytochrome *c*.

Since the coding sequence of the Bax gene contains a G_8_ track, it is particularly vulnerable to mutation in cells that are defective in DNA mismatch repair (Zhang *et al*, 2000). The loss of Bax renders cells resistant to some cytotoxic agents (Zhang *et al*, 2000) and blocks apoptosis mediated by the mitochondrial pathway (LeBlanc *et al*, 2002; Nutt *et al*, 2002). It also favours tumorigenesis (Ionov *et al*, 2000). On the other hand, in mouse embryonic fibroblasts (MEFs), the additional loss of the closely related proapoptotic protein Bak is required to block apoptotic cell death caused by a variety of death-inducing stimuli, including BH3-only proapoptotic proteins Bid, Bim or Bad (Wei *et al*, 2001; Zong *et al*, 2001).

Photodynamic therapy (PDT) is a novel treatment for cancer and other abnormal tissues that employs a photosensitiser and visible light to produce singlet oxygen and other reactive oxygen species (Weishaupt *et al*, 1976; Moan and Berg, 1992) that lead to subcellular damage at sites where the photosensitiser accumulates (Peng *et al*, 1996). PDT is an efficient inducer of apoptosis, with the initiating reactions dependent upon the preferential sites of photosensitiser localisation. Many of the commonly employed photosensitisers accumulate in the mitochondria. Like the protein kinase inhibitor staurosporine (STS), PDT with mitochondrion-damaging photosensitisers induces rapid apoptosis through activation of the mitochondrial pathway of apoptosis. This includes cytochrome *c* release, caspase activation, PARP cleavage, chromatin condensation and DNA fragmentation (Granville *et al*, 1998, 1999b; He *et al*, 1998; Kessel and Luo, 1998; Kim *et al*, 1999; Varnes *et al*, 1999; Chiu *et al*, 2001; Chiu and Oleinick, 2001; reviewed in Oleinick *et al* (2002)). The induction of apoptosis has also been observed in many tumours at early times following PDT at doses leading to tumour eradication (Zaidi *et al*, 1993; reviewed in Oleinick *et al* (2002)).

Bax undergoes a conformational change (exposure of an epitope detected by the 6A7 antibody) as well as translocation from cytosol to mitochondria immediately or shortly after PDT (Carthy *et al*, 1999; Granville *et al*, 1999b). The PDT-induced release of the mitochondrial apoptogenic proteins cytochrome *c* and Smac/DIABLO depends on Bax and is blocked in Bax-deficient human prostate cancer DU-145 cells (Usuda *et al*, 2002). Interestingly, stable overexpression of Bcl-2 can lead to the upregulation of Bax and increased sensitivity to PDT (Kim *et al*, 1999; Srivastava *et al*, 2001). However, other investigators have not found elevated Bax expression in cells stably transfected with Bcl-2 (He *et al*, 1996; Granville *et al*, 1999a).

Here, we report studies of the role of Bax in apoptosis and cell killing caused by PDT. Our data show that Bax is essential for the mitochondrial pathway of apoptosis induced either by PDT or STS. In its absence, downstream events of the mitochondrial pathway, including cytochrome *c* release, loss of mitochondrial membrane potential, caspase activation and chromatin condensation and fragmentation, are blocked. However, since cells deficient in Bax remain as photosensitive as Bax-proficient cells, we conclude that the commitment to cell death is likely determined before the step of Bax activation and cytochrome *c* release or independent of them.

## MATERIALS AND METHODS

### Cell culture and photodynamic treatment

The human prostate cancer cell line DU-145 was grown in Dulbecco's modified Eagle's medium, and the human breast cancer cell line MCF-7c3 was cultured in RPMI 1640 medium. Both media were supplemented with 10% fetal bovine serum, 2 mM L-glutamine and antibiotics. MCF-7c3 cells express a stably transfected human procaspase-3 (Xue *et al*, 2001b). The phthalocyanine photosensitiser Pc 4 (HOSiPcOSi(CH_3_)_2_(CH_2_)_3_N(CH_3_)_2_) was supplied by Dr Malcolm E Kenney of the Department of Chemistry, CWRU, and used as a 0.5 mM stock solution in dimethyl formamide. An aliquot of Pc 4 was added to the culture medium to give a final concentration of 200 nM, 16–18 h before light exposure. The light source was a light-emitting diode array (EFOS, Mississauga, Ontario, Canada; *λ*_max_ 670–675 nm). Both MCF-7c3 and DU-145 cells were irradiated with a fluence of 200 mJ cm^−2^ at a fluence rate of 1.0 mW cm^−2^. This dose of PDT causes about 90% killing of these cells, as determined by clonogenic assay. Irradiation was carried out at room temperature and was followed by incubation of the cultures in the dark for various periods of time before harvest.

### Determination of cell viability

Two methods were used to monitor cell viability. In the propidium iodide exclusion assay, cells were stained with 10 *μ*g ml^−1^ propidium iodide and 5 *μ*g ml^−1^ Hoechst 33342 in PBS and examined by fluorescence microscopy. For determination of the loss of clonogenicity, cells were collected from the monolayers with trypsin immediately after PDT and plated for colony formation (Xue *et al*, 2001b).

### Nuclear staining for detection of apoptotic cells

Cells grown on coverslips were fixed in PBS containing 3.7% formaldehyde. Cellular DNA was stained with 1–5 *μ*g ml^−1^ Hoechst 33342 and examined by fluorescence microscopy. Apoptotic cells were identified by characteristic features of their nuclei: condensation, margination and fragmentation of the chromatin. At least 200 cells were counted from each sample, and the yield of apoptotic cells was expressed as the percentage of the total population. Since detached cells, which are enriched in apoptotic cells, were not included in this measurement, the estimated percentage of apoptosis determined here is a minimal estimation of the true apoptosis levels.

### Fluorescence immunocytochemistry

Cells grown on coverslips were stained as described (Chiu *et al*, 2001; Chiu and Oleinick, 2001) with minor modifications. After fixation in 3% formaldehyde for 30 min on ice, cells were treated with 0.1% Triton X-100 in PBS for 10 min at room temperature. After blocking in IFA buffer (PBS containing 1% bovine serum albumin, 0.1% Tween 20), the coverslips were incubated with the primary antibody in IFA buffer followed by incubation in IFA buffer containing 0.5 *μ*g ml^−1^ Hoechst 33342 and the secondary antibody conjugated to Texas red (Vector Laboratories). All incubations were for 1 h at room temperature. Following thorough washing, the coverslips were mounted on slides with mounting medium (Kirkegaard & Perry Laboratories) and examined with a Leitz fluorescence microscope. Images were photographed with a Spot RT digital camera. The antibodies used in this study were mouse anti-cytochrome *c* (1 : 300 dilution, clone 6H2.B4, PharMingen) and rabbit anti-Bax (N1-20, Santa Cruz, 1 : 800 dilution).

### Measurement of mitochondrial membrane potential

Changes in ΔΨ_m_ were monitored by the uptake of JC-1, as previously described (Chiu and Oleinick, 2001). JC-1 (5,5′,6,6′-tetrachloro-1,1′,3,3′-tetraethylbenzimidazolyl-carbocyanine iodide) was supplied by Molecular Probes and dissolved in DMSO to produce a 1 mg ml^−1^ stock solution. Different aliquots of the cells were incubated at 37°C in culture medium containing 10 *μ*g ml^−1^ JC-1 for different 30-min periods starting immediately after light exposure until 2.5 h after PDT; that is, period 1, 0–30 min post-PDT; period 2, 30–60 min post-PDT, etc. Samples were washed once with PBS and examined for red–orange fluorescence (580 nm).

### Measurement of caspase 3 activity

Samples were prepared and assayed for caspase-3-like activity as described (Varnes *et al*, 1999). Briefly, approximately 2 × 10^6^ cells were collected by scraping with a rubber policeman and suspended in 120 *μ*l lysis buffer (20 mM HEPES, 10 mM KCl, 0.5% Triton X-100, 1 mM EDTA, 1 mM EGTA, 1 mM phenylmethylsulphonyl fluoride, 100 *μ*M pepstatin and 100 *μ*M leupeptin A, pH 7.4). Following sonication, aliquots containing 50 *μ*g protein were incubated with 50 *μ*M DEVD-AMC (BIOMOL) in 60 *μ*l of reaction buffer (25 mM HEPES, 10% sucrose, 0.1% CHAPS, 1 mM EGTA, 1 mM EDTA, 5 mM dithiothreitol, 1 mM phenylmethylsulphonyl fluoride, 100 *μ*M pepstatin, 100 *μ*M leupeptin, pH 7.4) for 1 h at 37°C. The released fluorescence was measured in a Perkin-Elmer LS50 fluorometer (*λ*_ex_, 380 nm; *λ*_em_, 460 nm).

### Cell transfection and Bax antisense (AS) treatment

DU-145 cells were grown in six-well plates at 3–5 × 10^5^ cells well^−1^ and were transfected with plasmid pcDNA3.Bax (a generous gift from Dr Minh Lam, CWRU Comprehensive Cancer Center) using Lipofectamine reagent (Life Technologies) according to the manufacturer's protocol. At 16–20 h after transfection, the medium was removed and replaced with a fresh medium containing Pc 4, and incubation was continued for an additional 2 h before irradiation. For Bax AS treatment, MCF-7c3 cells were grown either in six-well plates or in 60-mm Petri dishes; cells were transfected with 1 *μ*M Bax AS or scrambled phosphorothioate oligonucleotides using Lipofectamine. The transfected cultures were maintained for up to 4 days.

The 20-mer Bax AS and scrambled DNA oligonucleotides with a phosphorothioate backbone were synthesised and purified by high-pressure liquid chromatography (BIOSOURCE International, Camarillo, CA, USA). The 3′-oligonucleotides were biotinylated to facilitate the monitoring of intracellular uptake. The sequences employed were as follows: AS 1: 5′-TGCTCCCGGACCCGTCCAT-3′ (Gillardon *et al*, 1996); scrambled 1: 5′-TCATCGCTGGTAGAACACCT-3′; AS 2: 5′-TCGATCCTGGATGAAACCCT-3′ (Dibbert *et al*, 1999); scrambled 2: 5′-TGGTCCCGCTCCCGCCACAT-3′.

### Flow cytometry

Cells collected from cultures by trypsinisation were fixed with 1% formaldehyde (in PBS) for 15 min on ice. After permeabilisation with 0.1% Triton X-100, the cells were washed once with PBS and stained with propidium iodide (25 *μ*g ml^−1^) for 30 min before subjecting them to flow cytometric analysis. The stained samples were analysed on an EPICS ESP flow cytometer (Coulter Corp.) using the embedded instrument software.

### Western blot analysis

Cells were lysed, sonicated and boiled in protein gel buffer (50 mM Tris-HCl, pH 6.8, 1% SDS, 1% mercaptoethanol and 5% glycerol). The protein content of the lysate was measured using the BCA protein assay reagent (Pierce) and aliquots of 20 *μ*g protein were analysed on 12% SDS–PAGE gels. After transferring protein onto PVDF membranes, the proteins were probed with polyclonal anti-Bax (N-20, Santa Cruz) or anti-Bak (G-23, Santa Cruz). The immune complexes were detected by enhanced chemiluminescence system (Amersham).

## RESULTS

### Bax is translocated from the cytosol to mitochondria during PDT-induced apoptosis of MCF-7c3 cells

**Figure 1:**
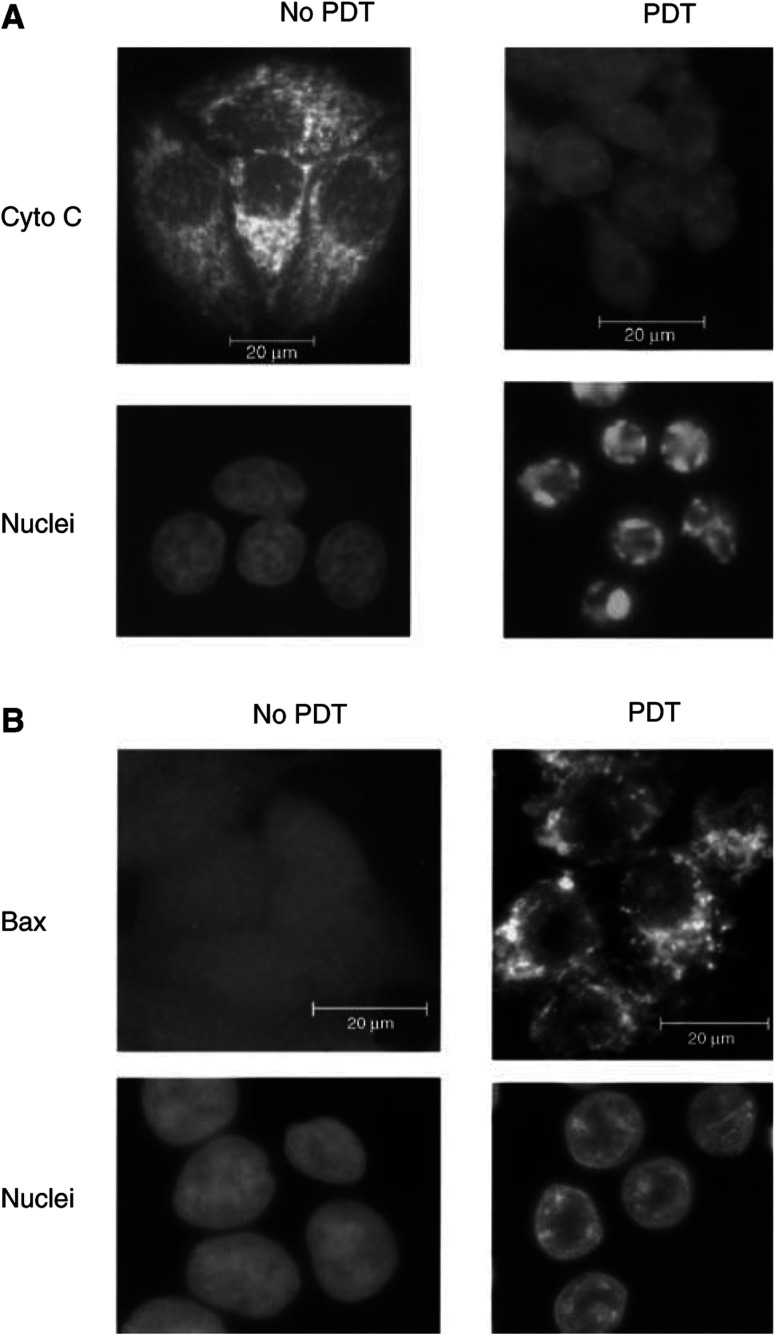
Redistribution of cytochrome *c* and Bax during PDT-induced apoptosis in MCF-7c3 cells. Fluorescence micrographs of immunocytochemically stained cytochrome *c* (**A**) or Bax (**B**) from untreated cells (left) or from cells 3 or 5 h after PDT (right). Nuclear DNA was stained with Hoechst 33342.

Pc 4-PDT, at a dose resulting in about 90% loss of clonogenicity (LD_90_ dose), induces rapid apoptosis in many cell lines through the activation of the mitochondrial (intrinsic) pathway (He *et al*, 1998; Chiu *et al*, 2002; Chiu and Oleinick, 2001; Lam *et al*, 2001; Usuda *et al*, 2002; Xue *et al*, 2001b). In many systems, Bax is required for the release of the mitochondrial intermembrane proteins and the induction of apoptosis (Finucane *et al*, 1999; Kim *et al*, 2001; Li *et al*, 2001). To assess the role of Bax in this pathway in the case of Pc 4-PDT, we first studied the relationship between Bax activation (translocation from cytosol to mitochondria) and an early step of the pathway, cytochrome *c* release, by immunocytochemistry in MCF-7c3 cells following a PDT dose of 200 nM Pc 4 and 200 mJ cm^−1^ (LD_90_ dose). Figure 1A

displays cytochrome *c* staining from untreated and PDT-treated apoptotic cells. Similar to our previous reports (Chiu *et al*, 2001; Chiu and Oleinick, 2001; Lam *et al*, 2001; Xue *et al*, 2003b), before PDT cytochrome *c* was confined to mitochondria, as shown by the perinuclear, punctate staining pattern, but 5 h after PDT, the staining pattern was diffuse throughout the cell, as a result of the release of cytochrome *c* from the mitochondria into the cytosol. PDT-treated cells also showed features of apoptotic morphology at this time. In contrast, Bax staining showed the inverse sequence (Figure 1B), that is, a diffuse pattern for control cells and a punctate pattern for apoptotic cells, consistent with the translocation of Bax from the cytosol to mitochondria during apoptosis, as demonstrated previously during apoptosis induced by a variety of stimuli (e.g., Wolter *et al*, 1997). A time-course study showed that the onset of Bax translocation and cytochrome *c* release occurs at 1 h after PDT, and the processes go to completion within the next 1–2 h. Since the maximal activation of caspase and nuclear apoptosis do not occur until 4 h after PDT in these cells, when cytochrome *c* has been completely released from the mitochondria (Xue *et al*, 2001b), the observations of Figure 1B indicate that translocation of Bax is an early event of PDT-induced apoptosis.

### Suppression of Bax expression with Bax AS oligonucleotides suppresses apoptosis induced by PDT or STS

**Figure 2:**
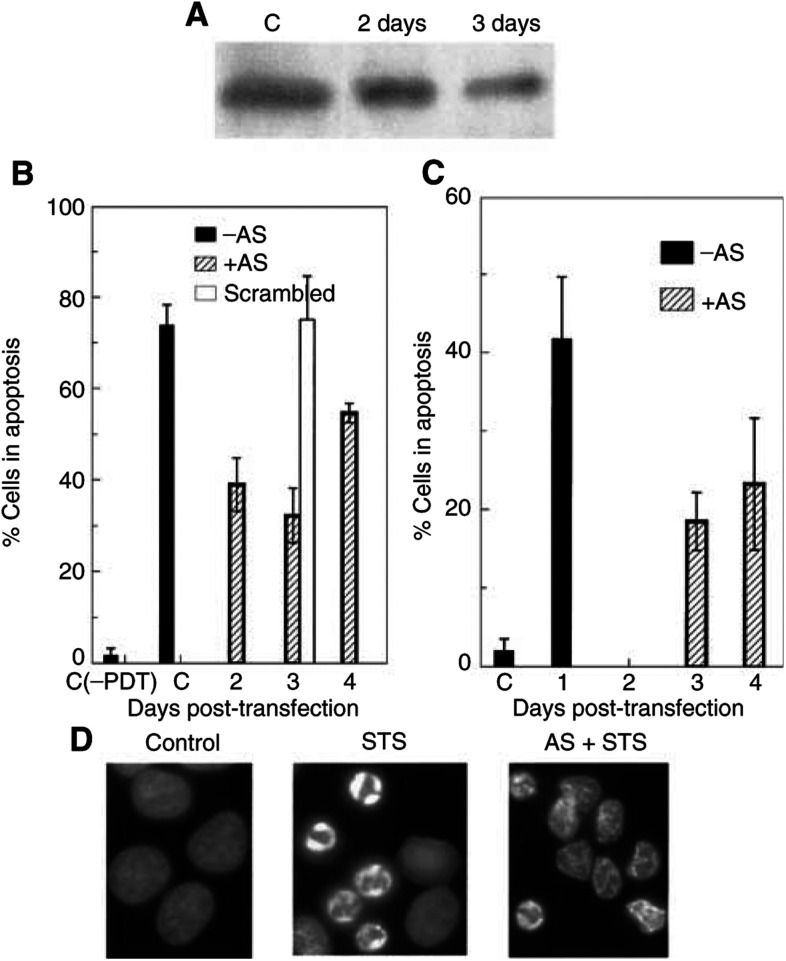
Suppression of Bax expression protects MCF-7c3 cells from PDT-induced apoptosis. (**A**) Western blot analysis of the levels of Bax after treatment with Bax AS for 2 or 3 days. (**B**, **C**) Levels of apoptosis induced by 1 *μ*M STS (**B**) or PDT (**C**) in MCF-7c3 cells transfected with Bax-AS or scrambled sequences. At 1–4 days after transfection, cells were exposed to PDT or STS, and 6 h later, cells were stained with Hoechst 33342 and apoptotic cells were counted. (**D**) Nuclear morphology of Bax AS-treated MCF-7c3 cells 6 h after STS treatment.

To determine the role of Bax in PDT-induced apoptosis, MCF-7c3 cells were treated with Bax AS to suppress Bax expression. Two Bax AS oligonucleotide sequences, previously reported to suppress Bax expression (Gillardon *et al*, 1996; Dibbert *et al*, 1999), were used. Since no significant difference was found between the two sequences with regard to the suppression of either Bax protein level or apoptosis, the results were pooled, and in later experiments only AS 1 was used. Although greater than 90% of cells exposed to the transfection procedure were positive for uptake of the AS oligonucleotides, as monitored by the staining of biotin-tagged AS oligonucleotides, only partial suppression of Bax expression was found upon Western blot analysis (Figure 2A

). The decrease in Bax expression by AS, however, was time-dependent, with the maximum suppression occurring ∼72 h after transfection. Increasing the AS oligonucleotide concentration from 1 to 5 *μ*M did not enhance suppression (data not shown). However, despite incomplete suppression of Bax protein by the Bax-AS, apoptosis induced by either PDT or 1 *μ*M STS was clearly inhibited (Figure 2B,C). There was a rough correlation between the inhibition of apoptosis and decrease in cellular Bax; that is, both measures were maximal 3 days after transfection, at which time the level of apoptosis was half that of cells exposed to PDT or STS in the absence of AS. The suppression of apoptosis by AS was specific, since treatment with scrambled oligonucleotides caused no significant effect on either Bax expression or apoptosis induction. It is interesting that the suppression of PDT- or STS-induced apoptosis in AS-treated cells was accompanied by the appearance of a large number of cells with abnormal nuclear morphology (Figure 2D). The abnormal nuclei were smaller and more brightly stained by Hoechst 33342 than normal interphase nuclei, perhaps resulting from partial shrinkage of the cells. Abnormal nuclei with similar morphology were also found in Bax-negative DU-145 cells after these treatments (see below).

### PDT-induced apoptosis is blocked in Bax-negative DU-145 cells

**Figure 3:**
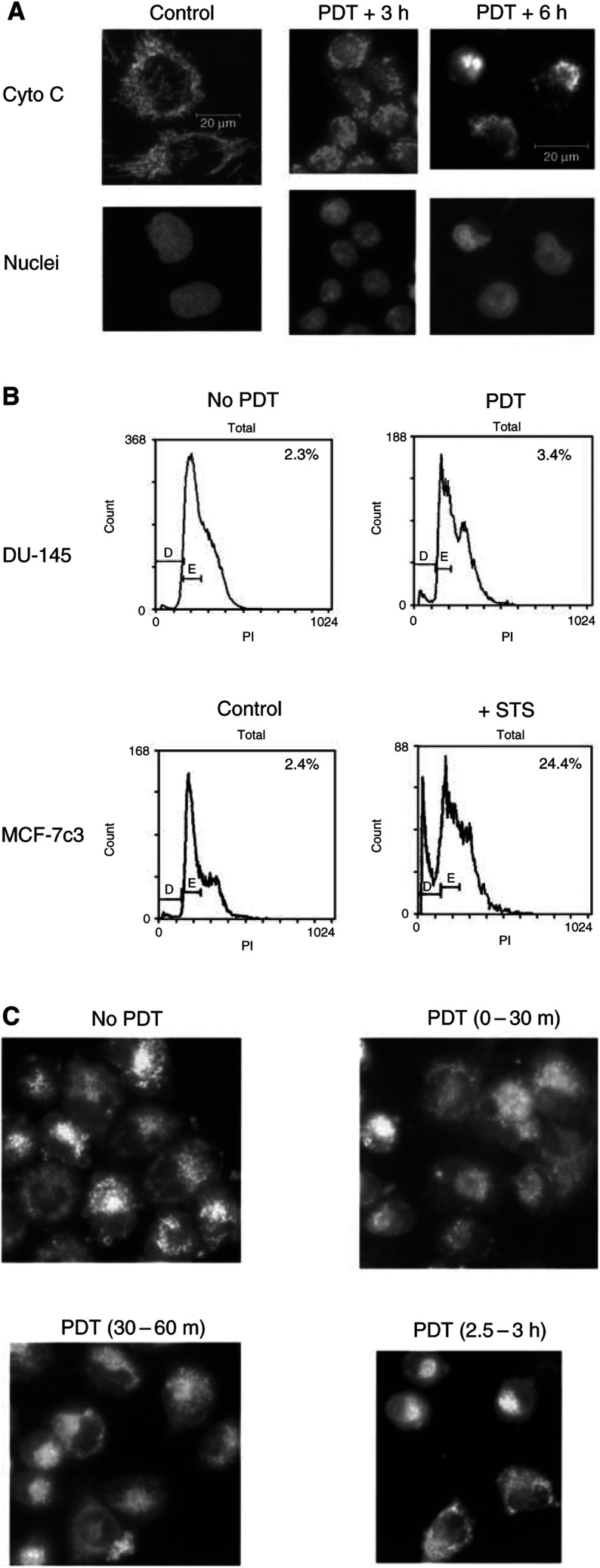
PDT fails to induce cytochrome *c* release, dissipation of mitochondrial membrane potential and apoptosis in DU-145 cells. (**A**) Fluorescence micrographs of immunocytochemically stained cytochrome *c* in untreated cells or in cells 3–6 h after PDT. Nuclear DNA was stained with Hoechst 33342. (**B**) Flow cytometric determination of the percentage of DU-145 (top) or MCF-7c3 (bottom) cells with less than the G1 content of DNA. DU-145 cells were either untreated (left) or exposed to PDT and recovered 6 h later (right). MCF-7c3 cells were either untreated (left) or exposed to 1 *μ*M STS for 16 h (right). Cells were stained with propidium iodide and analysed by flow cytometry. The percentage of apoptotic cells with a sub-G1 DNA content (Gate D) is given. (**C**) Fluorescence micrographs of DU-145 cells stained with JC-1 for 30 min at various intervals after PDT.

To determine whether Bax is the sole proapoptotic Bcl-2 family protein essential for PDT-induced apoptosis, the Bax-negative human prostate cancer cell line DU-145 was studied. The absence of Bax in these cells was confirmed by Western blot analysis (data not shown). When the cells were treated with an LD_90_ dose of PDT (200 nM Pc 4 and 200 mJ cm^−2^ red light), cell shrinkage was observed within 1 h of treatment. However, Hoechst 33342-stained nuclei from cells collected 5–20 h following PDT were characterised by the absence of the normal features of apoptosis, such as chromatin condensation, margination or fragmentation (Figure 3A

). Instead, the nuclei displayed a unique crescent shape that appears to result from the folding and collapse of the nuclear contents. The blockage of PDT-induced apoptosis in these Bax-deficient cells was further supported by the failure to detect a significant increase in the fraction of cells with sub-G1 DNA content by flow cytometry (Figure 3B). A similar blockage of apoptosis was found in DU-145 cells 6 h following treatment with 1 *μ*M STS (data not shown). In contrast, more than 20% of the Bax-replete MCF-7c3 cells contained a sub-G1 level of DNA after the same treatment with 1 *μ*M STS (Figure 3B).

### Pc 4-PDT caused neither the release of cytochrome *c* from mitochondria nor the loss of mitochondrial membrane potential in DU-145 cells

Since the translocation of Bax from the cytosol to mitochondria has been demonstrated to be essential for the release of cytochrome *c* in response to a variety of apoptotic stimuli, we next examined the redistribution of cytochrome *c* following Pc 4-PDT in DU-145 cells. As shown in Figure 3A, immunocytochemical staining of cytochrome *c* from untreated cells reveals a perinuclear and punctate pattern, as described for MCF-7c3 cells. The stained cytochrome *c* of PDT-treated cells maintains the same punctate pattern at 3 h, but becomes aggregated and clustered in a few cellular sites at later hours as cell shrinkage occurs (Figure 3A). The cytochrome *c* staining pattern is markedly different from the diffuse pattern that results from the redistribution of cytochrome *c* from the mitochondria to the cytoplasm shown above, indicating that cytochrome *c* was retained in the mitochondria of DU-145 cells. Smac, another protein of the mitochondrial intermembrane space, is also known to be released from mitochondria during apoptosis; release of Smac leads to caspase activation through suppression of caspase inhibitors, such as XIAP (Du *et al*, 2000). Like cytochrome *c*, Smac remained associated with mitochondria following treatment of DU-145 cells with Pc 4-PDT (Usuda *et al*, 2002).

The dissipation of the mitochondrial membrane potential (ΔΨ_m_) following PDT was monitored by the uptake of the potential-sensitive indicator dye JC-1. Despite cell shrinkage, as well as clustering and alteration in the cellular distribution of mitochondria, there was no significant decrease in the uptake of JC-1 by DU-145 cell mitochondria for up to 3 h following PDT (Figure 3C). However, because the uptake of JC-1 was monitored by recording the amount of dye accumulated in 30-min periods, the possibility of a transient depression of uptake without a significant effect on overall uptake cannot be ruled out. Moreover, consistent with the lack of cytochrome *c* release and apoptosis following PDT or STS, these treatments failed to induce activation of caspase-3-like proteases (DEVDase) in DU-145 cells (data not shown).

### Expression of Bax in DU-145 cells restores apoptosis

**Figure 4:**
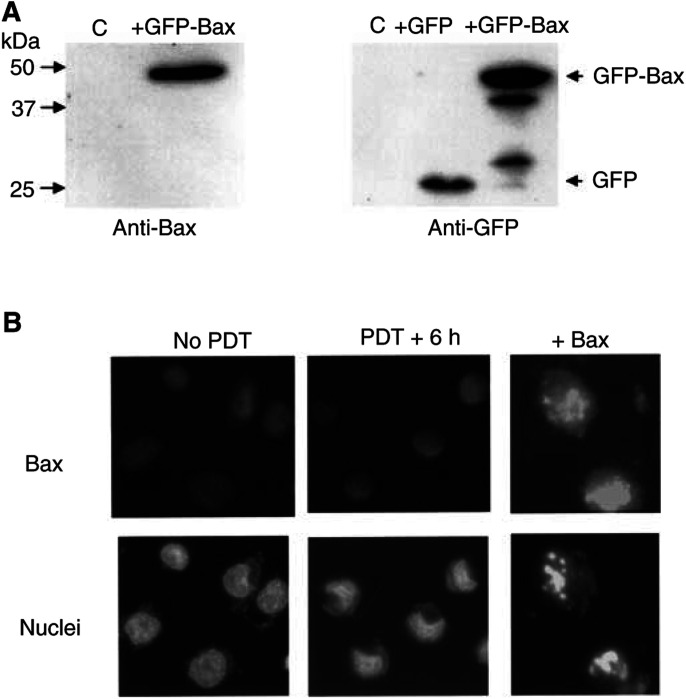
Expression of Bax in DU-145 cells restores apoptosis. (**A**) Western blot analysis of the levels of Bax in DU-145 cells 20 h after transfection with a plasmid encoding GFP-Bax or GFP. Blots were probed with anti-Bax (left panel) or anti-GFP (right). (**B**) Immunocytochemical detection of Bax expression (upper panels) and nuclear morphology (lower panels) of DU-145 cells. Nontransfected cells were either untreated (left panels) or PDT-treated and examined 6 h later (middle panels). Other cells were examined 20 h after transfection with a Bax-expression plasmid.

DU-145 cells are known to be mismatch repair defective (Chen *et al*, 2001), so that in addition to loss of Bax, mutations in one or more additional components of the apoptosis pathway may also be present. To evaluate the importance of Bax deficiency, the cells were transfected with an expression plasmid encoding the Bax-GFP fusion protein. At 20 h after the transfection, 24±8% of the cells were positive for GFP expression, and 13±3% of them contained less than the G1 DNA content, as detected by flow cytometry. The production of a high level of Bax in the transfected cells was confirmed by Western blot analysis (Figure 4A

). Transfection also caused about 10% of the cells to detach from the monolayer during the incubation, of which 45±12% displayed morphological features of apoptosis (Figure 4B) as well as sub-G1 DNA content (data not shown). Furthermore, immunocytochemical staining of Bax revealed that while the majority of attached cells contained no Bax, many of the detached apoptotic cells stained intensely for Bax in a manner that was perinuclear and punctate, an indication of the presence of a high level of Bax and its association with mitochondria (Figure 4B). Thus, the intrinsic apoptotic machinery is intact in DU-145 cells, and apoptosis can be restored in these cells by the expression of Bax. Attempts to select stable Bax-transfected cells, however, were not successful, probably because the high level of Bax expression causes apoptosis in all of the transfected cells.

### Bax-negative DU-145 cells were sensitive to PDT

**Figure 5:**
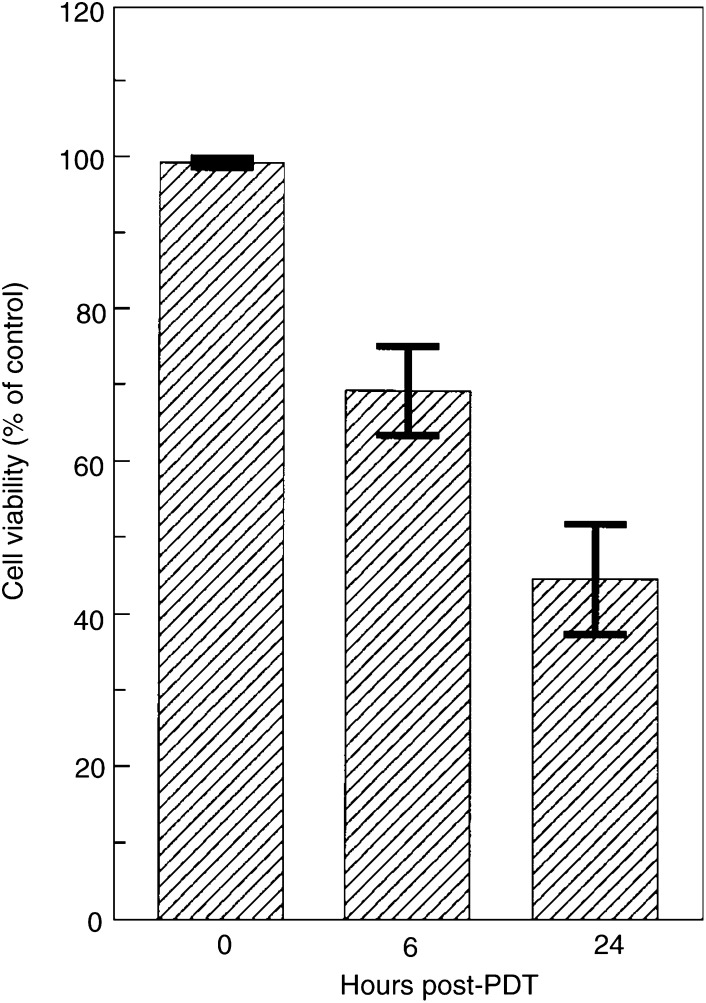
Viability of DU-145 cells 0, 6 or 24 h after PDT, as determined by propidium iodide exclusion assay. The data represent the mean±s.d. of three experiments.

Since Bax-negative DU-145 cells are resistant to apoptosis after PDT, and Bax-deficient cells have been shown to be resistant to agents such as indomethacin and sulindac (Zhang *et al*, 2000), we tested whether these cells are resistant to killing by PDT. Figure 5

presents the results of the measurement of cell viability by the ability to exclude propidium iodide. It shows that the fraction of cells that has lost plasma membrane integrity increased with time after PDT, and by 24 h >50% of the cells had lost viability. The overall cell killing was monitored by clonogenic assay, and our previous data on Bax-replete MCF-7c3 cells are included for comparison (Table 1

**Table 1:** Clonogenic survival of MCF-7c3 and DU-145 cells after treatment with PDT or STS

	**% Survival**	
**Treatment**	**DU-145**	**MCF-7c3**
PDT	5.5±2.2	13.4±5.5^a^
Staurosporine	12.8±2.2	0.45±0.01^b^

Cells were treated with either PDT (200 nM Pc 4 and 200 mJ cm^−2^) or with 1 *μ*M STS (6 h) and then plated for colony formation. Each datum is the mean±s.d. of results from triplicates of at least two independent experiments.

a Data from Xue *et al* (2001b).

b Data from Xue *et al* (2003a).

). As shown in the table, 95% of the treated DU-145 cells were unable to form colonies after the same PDT dose that caused 87% loss of clonogenicity of Bax-replete MCF-7c3 cells. Therefore, the mutation that prevents Bax expression does not render the cells more resistant to PDT. Table 1 also shows that DU-145 cells are also sensitive to STS. However, they were some 28-fold more resistant than MCF-7c3 cells to the same dose of STS.

## DISCUSSION

The present report shows that the resistance of DU-145 cells to the induction of apoptosis by PDT or STS is not due to a defect in the intrinsic apoptotic machinery, but rather due to the absence of the proapoptotic Bcl-2 homologue Bax, since apoptosis is restored by the expression of Bax in these cells. This conclusion is consistent with a recent finding by Li *et al* (2001) that the overexpression of Bax mediated by an adenovirus vector in DU-145 cells leads to apoptotic cell death, as demonstrated by the release of cytochrome *c*, activation of caspases-3, -9 and -7 and DNA fragmentation. These data, together with the findings of the early translocation of Bax from the cytosol to mitochondria following PDT in Bax-replete MCF-7c3 cells, as well as the suppression of apoptosis in these cells upon treatment with Bax-AS, clearly establish that Bax is the sole proapoptotic Bcl-2 family member essential for apoptosis in these human cancer cells in response to the two test inducers. A similar stringent requirement for Bax to activate the intrinsic pathway of apoptosis has been demonstrated for human colon cancer HCT116 cells (Zhang *et al*, 2000; LeBlanc *et al*, 2002). In contrast, Bax does not appear to be required for the extrinsic pathway of apoptosis, since death receptor activating agents are able to induce apoptosis in Bax-negative DU-145 (Chatterjee *et al*, 2001). This situation is in stark contrast to requirements for apoptosis in murine embryonic fibroblasts, in which Bax and its close homologue Bak have redundant functions, because both genes must be deleted for apoptosis to be blocked (Wei *et al*, 2001).

In the absence of Bax, the downstream events of the mitochondrial pathway of apoptosis, such as the release of cytochrome *c*, dissipation of the mitochondrial membrane potential, caspase activation, and chromatin condensation and fragmentation, are completely blocked after PDT. Thus, the present data confirm that PDT induces apoptosis through this signalling pathway. However, although mitochondria have been shown to be a prime target of PDT with certain photosensitisers (Kessel and Luo, 1998), and PDT causes photodamage to mitochondrion-bound proteins, in particular Bcl-2 (Xue *et al*, 2001a) and Bcl-xL (Xue *et al*, 2003a), and to mitochondrial and endoplasmic reticulum membrane structure (Grebenova *et al*, 2003), the present data suggest that the photodamage is insufficient to cause the release of cytochrome *c* spontaneously through the outer mitochondrial membrane. The release needs the participation of Bax. This finding is consistent with the conclusion (Zong *et al*, 2001) that Bax or Bak is needed as an effector of apoptosis, even when antiapoptotic Bcl-2 proteins are neutralised by the overexpression of BH-3 peptide. The requirement that Bax must be activated and migrate to the mitochondria for the release of cytochrome *c* would predict (a) a delay in the cytochrome *c* release process after PDT, as we previously observed in mouse lymphoma LY-R cells and in human tumour A431 and MCF-7 cells (Chiu *et al*, 2001; Chiu and Oleinick, 2001; Lam *et al*, 2001; Xue *et al*, 2001b) and (b) temperature dependence of the processes of Bax migration and cytochrome *c* release (Kessel and Castelli, 2001). In contrast, the prediction is inconsistent with earlier reports of an immediate release of cytochrome *c* following PDT (Granville *et al*, 1998; Kessel and Luo, 1998).

In spite of the resistance of Bax-negative DU-145 cells to apoptosis induction by PDT or STS, the cells remain as sensitive to killing by PDT as are Bax-replete MCF-7c3 cells, as judged by the loss of clonogenicity. The results indicate that the commitment to cell death is independent of the execution of apoptosis. A similar result was obtained with caspase-3-deficient MCF-7v cells, wherein stable expression of caspase-3 (MCF-7c3) restored apoptosis capability and enhanced the rate of cell death, but had no significant influence on overall cell killing by PDT, as determined by clonogenic assay (Xue *et al*, 2001b). Since the release of cytochrome *c* after PDT proceeds normally in MCF-7 cells, whether or not they contain functional caspase-3, the observation supports the proposal (Green and Amarante-Mendes, 1998) that the step of cytochrome *c* release is the point-of-no-return for cell death. Furthermore, our previous study on apoptotic cell death induced by PDT in mouse lymphoma cells showed a good correlation in dose response between the fraction of cells killed by apoptosis and the fraction of cells that release cytochrome *c* after PDT (Chiu *et al*, 2001). The release of cytochrome *c* can either trigger caspase activation or cause mitochondrial dysfunction. Either way will eventually lead to cell death. The present observation of cell killing in the absence of cytochrome *c* release in Bax-negative cells suggests that this hypothesis needs modification. We propose that the commitment to cell death occurs at or before Bax activation, which includes its migration and integration to the mitochondria.

Bax mutation is common in tumours, because (a) the Bax gene contains a G_8_ mononucleotide track and hence is prone to mutation, particularly in cells defective in mismatch repair, and (b) the inactivation of Bax confers on the cells a survival advantage and promotes tumour progression (Ionov *et al*, 2000). Indeed, more than half of colon tumours of the MMR type contain Bax mutations (Rampino *et al*, 1997). Moreover, patients with Bax mutations also have a poor prognosis (Ionov *et al*, 2000). Interestingly, our data show that Bax-negative DU-145 cells are more resistant to STS than are the Bax-replete MCF-7c3 cells, whereas cell killing by PDT is not compromised by the absence of Bax (Table 1). This observation suggests that PDT may have an advantage over other therapies in the treatment of tumours with Bax mutations.
